# Cost‐Effective 3D‐Printed Bionic Hydrogel Evaporator for Stable Solar Desalination

**DOI:** 10.1002/advs.202308665

**Published:** 2024-02-11

**Authors:** Shuang Zhang, Meng Li, Chaorui Jiang, Dandan Zhu, Zhihui Zhang

**Affiliations:** ^1^ Key Laboratory of Bionic Engineering Ministry of Education College of Biological and Agricultural Engineering Jilin University No. 5988 Renmin Street Changchun 130022 P. R. China; ^2^ The State Key Laboratory of Supramolecular Structure and Materials College of Chemistry Jilin University No. 2699 Qianjin Street Changchun 130023 P. R. China

**Keywords:** 3D printed, bionic structures, cost‐effective, hydrogel evaporators, solar desalination

## Abstract

Solar desalination using hydrogel evaporators is an eco‐friendly, highly efficient means with natural sunlight for sustainable freshwater production. However, it remains challenging to develop a cost‐effective and scalable method to prepare salt‐resistant hydrogel evaporators for stable desalination. Here, inspired by tree transpiration and hierarchical porous structure, a 3D‐printed bionic hydrogel evaporator (3DP‐BHE) is designed for long‐term solar desalination. Commercialized activated carbon (AC) is introduced into biomass starch skeleton as a solar light absorber to build 3DP‐BHE in a cost‐effective fashion ($10.14 m^−2^ of total materials cost). The bionic tree leaf layer for 94.01% light absorption and timely vapor diffusion. The bionic tree trunk layer with 3D printed bimodal porous structure for water transfer, thermal isolation, and salt ions convection and diffusion. With the unique bionic structure, the 3DP‐BHE achieves a stable evaporation rate of 2.13 kg m^−2^ h^−1^ at ≈90.5% energy efficiency under one sun (1 kW m^−2^). During the 7‐day desalination of 10 wt.% brine, a steady evaporation rate of 1.98 kg m^−2^ h^−1^ is maintained with a record‐high cost‐effectiveness (195.3 g h^−1^ $^−1^) manner. This 3DP‐BHE will open significant opportunities for affordable solar desalination systems on multiple scales, from individual households to off‐grid communities.

## Graphical abstract

Tree‐inspired, 3D‐printed bionic hydrogel evaporator exhibits a stable evaporation rate of ≈1.98 kg m^−2^ h^−1^ during the 7‐days desalination of 10 wt.% NaCl brine under one sun illumination with outstanding cost‐effectiveness (195.3 g h^−1^ $^−1^). Sustainable and safe starting materials with scalable preparation methods show great advantages for multiple scales practical applications.

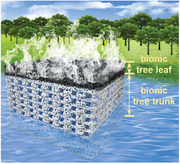

## 1 Introduction

Freshwater scarcity, which affects 3 billion people, is one of the major threats to the sustainable development of human societies due to global population growth, climate change, and the modernization of industry and agriculture.^[^ ^1^, ^2^ ^]^ Solar desalination based on vapor generation, which directly uses renewable and eco‐friendly natural light instead of fossil energy, is the next generation of water desalination technology with zero carbon emission.^[^ ^3^, ^4^, ^5^ ^]^ To date, various carbonized natural green flora evaporator has been developed.^[^ ^6^ ^]^ However, the evaporation rates of recently reported carbonized flora evaporators are less than 1.6 kg m^−2^ h^−1^ under one sun (1 kW m^−2^). One primary reason is that water desalination is an energy‐intensive process, and the diffused sunlight under natural conditions barely meets the energy demand for water evaporation.^[^ ^7^ ^]^ Hydrogel evaporators have remarkably high evaporation rates due to they can circumvent insufficient solar energy supply by altering the interaction between polymers and water molecules to reduce the vaporization enthalpy of water.^[^ ^8^, ^9^ ^]^ However, the ultrahigh evaporation rate causes dramatic salt accumulation on the evaporated surface, eventually inducing salt crystallization, reducing the evaporation rate and shortening the lifetime of the evaporator.^[^ ^10^ ^]^ Avoiding salt crystallization on the hydrogel surface during evaporation is a prerequisite for stable solar desalination.

Recently, salt resistance hydrogel evaporators have been designed to achieve stable and efficient evaporation rates using the strategies of local crystallization,^[^ ^11^ ^]^ ion rejection,^[^ ^12^, ^13^ ^]^ Janus design,^[^ ^14^, ^15^ ^]^ and diffusion and convection.^[^ ^16^, ^17^, ^18^, ^19^ ^]^ Among those strategies, diffusion and convection are the most widely applicable methods since they can be universally applicable to most hydrogels with feasible structures. Intrinsic ultralow diffusivity of salt in water (≈10^−9^ m^2^ s^−1^, compared to the diffusion rate of water vapor in air, 10^−5^ m^2^ s^−1^) and hydrogel high tortuosity make it difficult to achieve salt resistance by diffusion alone.^[^ ^20^ ^]^ Introducing convection by increasing the interconnectivity of the hydrogel network is essential.^[^ ^21^ ^]^ Porous hydrogel evaporators prepared by directional freezing^[^ ^22^ ^]^ or self‐assembled template (SAT) method^[^ ^23^ ^]^ were developed to synergize convection and diffusion to promote salt rejection. However, the porous structure reliability of dynamic water supply and salt convection is restricted when constructing large‐sized hydrogel evaporators for practical applications. Apart from the size limitation, expensive petroleum‐based raw materials fail to achieve wide‐scale application in economic water scarcity areas,^[^ ^24^ ^]^ toxic cross‐linkers offset the large benefit of eco‐friendly.^[^ ^25^, ^26^ ^]^ In the framework of the dual‐carbon concept, it is necessary to develop a low‐cost hydrogel evaporator with an environmentally friendly and scalable preparation method to achieve stable desalination for practical application in multiple scales, from individual households to off‐grid communities.

**Figure 1:**
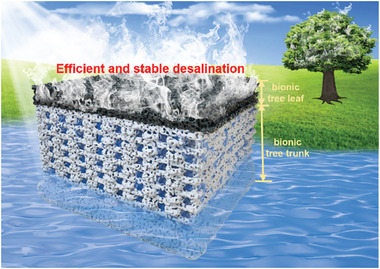
Schematic of the 3D‐printed bionic hydrogel evaporator (3DP‐BHE) for efficient and stable solar desalination. The top light‐absorbing layer is similar to a tree leaf for light absorption and vapor diffusion. The bottom layer with the bimodal porous structure is similar to a tree trunk for fast water uptake, effective thermal isolation, and timely salt ions diffusion and convection.

The tree with a hierarchical porous structure can transport water from the soil to the leaves during their photosynthesis process, simultaneously water vapor releases caused by transpiration.^[^ ^27^, ^28^ ^]^ It plays a vital role in the global hydrological cycle driven by solar and wind energy. Here, inspired by the tree leaf transpiration and trunk water transport porous structure, we develop a cost‐effective 3D‐printed bionic hydrogel evaporator (3DP‐BHE). The photothermal layer is similar to a tree leaf for 94.01% light absorption and rapid vapor diffusion. The bimodal porous structure, corporate with high degree interconnectivity wick channels formed by 3D printing and the inherent open microchannels in hydrogels, is similar to a tree trunk for fast water pumping, effective thermal isolation, and timely salt ions diffusion and convection (**Figure** **1**). With a low total material cost of $10.14 m^−2^, the evaporation rate as high as 2.13 kg m^−2^ h^−1^ with 90.5% solar‐to‐vapor efficiency is achieved by the 3DP‐BHE under one sun illumination. Most importantly, the 3DP‐BHE performed a steady evaporation rate of 1.98 kg m^−2^ h^−1^ and impressive cost‐effectiveness of 195.3 g h^−1^ $^−1^ in 10 wt.% brine during the 7‐day desalination. Sustainable and safe starting materials with scalable preparation methods of 3DP‐BHE show a great advantage for the potential solar desalination need.

## 2 Results and Discussion

### 2.1 Design Principles and Salt Resistance Mechanism of the 3D‐Printed Bionic Hydrogel Evaporator

For practical application, salt‐resistant hydrogel evaporators need to meet the following principles: scalability, low cost, environmentally friendly, and structure stable. First, 3D printing technology can fabricate highly interconnected pores structure with excellent customizability and repeatability. Direct ink writing 3D printing is an ideal method for large‐scale production of interconnectivity porous hydrogel evaporators, which can significantly reduce the time and capital costs involved in preparation. Second, widespread, cheaper and renewable natural biomass is the best choice for preparation hydrogel evaporators. Among them, pregelatinized starch with unique rheological properties is suitable for direct ink writing 3D printing.^[^ ^29^ ^]^ Furthermore, the presence of a large number of hydroxyl groups in the starch molecular chain enables the formation of stable hydrogels after several freeze‐thaw cycles, which also simplifies the gelation process after printing and avoids the introduction of other toxic components. AC was chosen for solar absorption to facilitate industrial production and reduce material costs.

**Figure 2:**
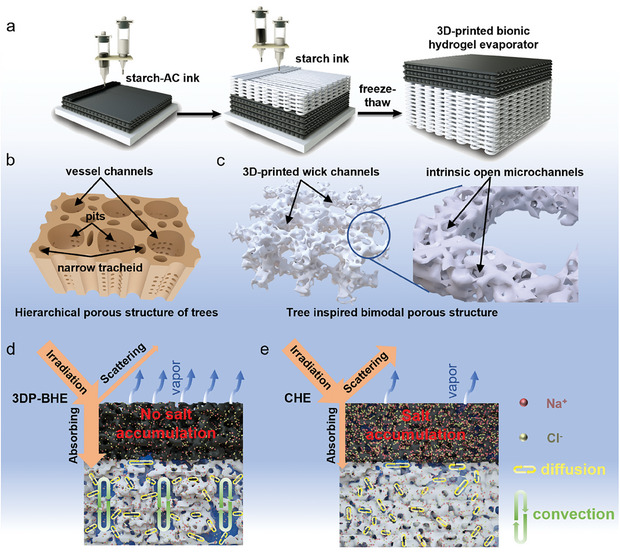
Schematic the fabrication and salt resistance mechanism of the 3DP‐BHE. a) Prepare process of the 3DP‐BHE. b) Hierarchical porous structure of trees. c) Bionic trunk with bimodal porous structure of 3DP‐BHE. Salts rejection mechanism of the d) 3DP‐BHE and e) CHE hydrogel evaporator.

The 3D‐printed bionic hydrogel evaporator preparation process is shown in **Figure** **2**a, tightly filled starch‐AC ink was first printed, and then starch ink was vertically printed on the top of the starch‐AC layer. After multiple freeze‐thaw cycles, the 3DP‐BHE was removed from the substrate and inverted. The initially printed starch‐AC serves as a bionic tree leaf for light absorption and vapor diffusion. The bottom starch layer with the bimodal porous structure serves as a bionic tree trunk, which mimics the hierarchical porous structure of the tree. As shown in Figure 2b, the hierarchical porous structure of the tree consists of larger vessel channels with diameters ranging from 180 to 390 µm and narrower tracheid channels with diameters ranging from 18 to 39 µm. Numerous micro‐sized pits (≈1‐2 µm) exist in the hierarchical porous structure cell walls.^[^ ^30^ ^]^ These structures are used to realize water transport and nutrient exchange. The 3D‐printed bimodal porous structure is corporate with high degree interconnectivity wick channels formed by 3D printing, mimicking the large vessel channels of trees, and the inherent open microchannels in hydrogels, mimicking the pits of trees (Figure 2c). This unique structure facilitates water transport and salt ions exchange.

During long‐term solar desalination, the concentration of brine in 3DP‐BHE will continue to increase. Due to the timely water transport in the wick channels, the salt concentration in the wick channels will be much lower than that in the hydrogel intrinsic microchannels, thereby creating an in‐plane concentration gradient. The concentration gradient leads to spontaneous salt diffusion from the microchannels to wick channels through the open porous of the hydrogel. The interconnectivity wick channels reduce the tortuosity of the salt transport channel and accelerate gravity‐driven salt diffusion and convection, ultimately avoiding the formation of salt crystals on the evaporator surface (Figure 2d). Compared with the conventional hydrogel evaporator (CHE), the high tortuosity hydrogel network limits convection and diffusion, leading to salt accumulating on evaporator surfaces, eventually blocking water transport channels and reducing sunlight absorption and vapor escape (Figure 2e).

### 2.2 Characterization of the 3DP‐BHE

**Figure 3:**
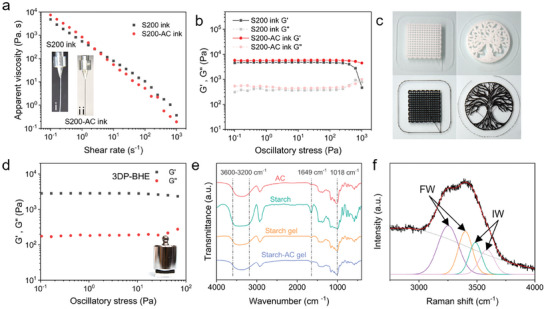
Characterization of the 3DP‐BHE. a) Apparent viscosity as a function of shear rate for S200 and S200‐AC ink, inset showing two inks smoothly extruded from a 300 µm nozzle. b) Storage modulus (G′) and loss modulus (G″) of S200 and S200‐AC ink as a function of oscillatory stress. c) Photographs showing that various patterns can be printed using S200 ink or S200‐AC ink by the extrusion‐based 3D printer. d) Storage modulus (G′) and loss modulus (G″) of the 3DP‐BHE as a function of oscillatory stress, inset showing hydrated 3DP‐BHE can withstand 100 g weights without deformation. e) FTIR spectra of pure AC, starch, starch gel and starch‐AC gel. f) Raman spectrum with fitting curves of the 3DP‐BHE.

Ink rheological properties play a crucial role in the direct ink writing 3D‐printed process. An ideal 3D printing ink should have shear‐thinning properties to ensure it can be smoothly and continuously extruded from a fine nozzle under appropriate pressure. After extrusion, the ink unloads shear stress to form a stable hydrogel filament. Finally, the hydrogel filament is stacked layer by layer to form a three‐dimensional structure.^[^ ^31^ ^]^ To confirm that the pregelatinized starch is suitable for direct ink writing 3D printing, the rheological properties of starch ink and starch‐AC ink were measured. The water transport layer ink with a starch concentration of 200 mg ml^−1^ was named S200 ink. The solar absorbing layer ink was prepared by adding 10 mg ml^−1^ AC as a solar absorber to 200 mg ml^−1^ starch solutions and labeled as S200‐AC ink. To improve the dispersion of the solar absorber in the hydrogel, AC particles with a diameter of 420 nm were hydrophilicity treated to make them uniformly dispersed in water (Figure S1, Supporting Information). The corresponding 3D‐printed bionic hydrogel evaporator prepared with the above inks was denoted as 3DP‐BHE. As shown in **Figure** **3**a, both S200 ink and S200‐AC ink display a non‐Newtonian behavior of shea‐thinning. With the shear rates increasing from 10^−1^ to 10^3^ s^−1^, S200‐AC ink viscosity dropped from ≈7500 to ≈ 60 Pa s. The S200 ink viscosity curves almost had the same trend. At low shear rates, both S200 ink and S200‐AC ink have high viscosity and can maintain a stable structure. The low viscosity associated with the high shear rate ensures that both two inks were smoothly extruded from a 300 µm diameter nozzle (inset in Figure 3a). Figure 3b displays the variation of storage modulus (G′) and loss modulus (G″) with oscillatory stress for S200 ink and S200‐AC ink. G′ is greater than G″ proves that both inks exhibit gel properties at low shear stress and ensures that they can keep their structure stable after printing.^[^ ^32^ ^]^ The storage modulus plateau value of S200‐AC ink (G' ≈5.7 × 10^3^ Pa) is higher than S200 ink (G' ≈4.5 × 10^3^ Pa). The stiffer nature of S200‐AC ink makes it more suitable as a bottom layer during the 3D printing process to bear the weight of S200 ink and retain its shape.^[^ ^33^ ^]^ At high oscillatory stress, the storage modulus of the ink decreases dramatically indicating that the ink structure is gradually destroyed and causes a yielding behavior. S200 ink and S200‐AC ink can print various sophisticated structures (Figure 3c). Figure 3d reveals the mechanical strength of the hydrogel evaporator. G′ is considerably higher than G″ confirms the cross‐linked polymeric skeleton of the 3DP‐BHE. Furthermore, the 3DP‐BHE with good mechanical strength can maintain its full shape during solar desalination (Figure 3d and Figure S2, Supporting Information).

The chemical composition of the 3DP‐BHE was analyzed by Fourier transform infrared (FTIR) spectroscopy (Figure 3e). The broad peaks located at 3200–3600 cm^−1^ are attributed to the stretching vibrations of hydroxyl groups and the peaks at 1649 and 1018 cm^−1^ correspond to the bending vibrations of the hydroxyl groups within starch and activated carbon. Comparing the infrared spectra of the hydrogels with pure starch and pure AC found that no new peaks appeared, confirming that no chemical reaction occurred between the AC and starch chains, revealing that the AC was incorporated into the starch system by intermolecular forces. The Raman spectra of water confined in the 3DP‐BHE proves the existence of free water (FW) and intermediate water (IW) (Figure 3f). The peaks at 3234 and 3390 cm^−1^ are related to the in‐phase and out‐of‐phase vibration modes of O─H in water that have four hydrogen bonds with surrounding molecules, respectively, indicating the presence of FW. The peaks at 3518 and 3631 cm^−1^ correspond to the symmetric and asymmetric stretching of IW with the weak hydrogen, respectively.^[^ ^9^ ^]^ These results indicate that IW is contained in the 3DP‐BHE, reduces the vaporization enthalpy of water and facilitates water evaporation.

### 2.3 Structure and Morphologies of the 3DP‐BHE

**Figure 4:**
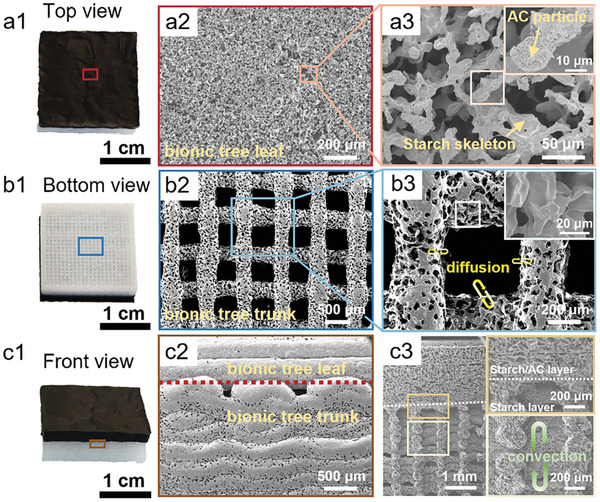
Morphologies of the 3DP‐BHE. a1) Upper layer of the 3DP‐BHE and a2), a3) SEM images of upper starch‐AC layer. b1) Bottom layer of the 3DP‐BHE and b2), b3) SEM images of bottom starch layer. c1) Front view of the 3DP‐BHE, c2) SEM image exhibiting the junction of starch layer and starch‐AC layer, c3) SEM images showing cross morphology of the 3DP‐BHE, which visualizes the bionic structure.

The 3DP‐BHE manufactured using direct ink writing 3D printing technology possesses both macroscopic and microscopic structures. The morphology of the 3DP‐BHE was characterized by scanning electron microscopy (SEM). **Figure** **4**a1 shows the bionic tree leaf layer of 3DP‐BHE with black AC as the solar absorber. It is composed of tightly bonded S200‐AC hydrogel filaments (Figure 4a2). Open micropores increase solar absorption and ensure water vapor unhindered diffuses during the solar vapor generation process (Figure 4a3). AC particles uniformly and densely cover on starch skeleton surface to absorb sunlight (Figure 4a3 inset). The bionic tree trunk of the 3DP‐BHE is presented in the bottom view (Figure 4b1). The bimodal porous structure is corporate with 400 µm wick channels formed by 3D printing (Figure 4b2) and the inherent sub‐10 µm open microchannels in hydrogel (Figure 4b3). Interconnection between wick channels and microchannels ensures timely water transport and rapid diffusion of salt ions (Figure S3, Supporting Information). Figure 4c, 1 presents the interface layer of 3DP‐BHE. The upper bionic tree leaf is strongly bound with the bottom bionic tree trunk, which ensures the 3DP‐BHE remains integrity during the solar evaporation process (Figure 4c, 2). The cross‐sectional morphology of 3DP‐BHE consists of compact stack multiple layers microfilaments, clearly exhibiting the bionic bimodal porous structure (Figure 4c, 3). The bionic tree trunk with abundant interconnectivity wick channels in the vertical direction reduces convective resistance and ensures expeditious ion convection.

### 2.4 Water Evaporation Performance of the 3DP‐BHE

**Figure 5:**
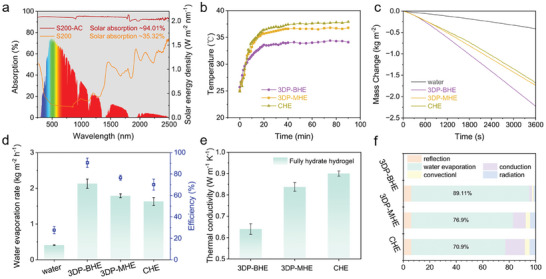
Performance of solar vapor generation based on hydrogel evaporators. a) UV–vis–NIR spectra of the 3DP‐BHE light‐absorbing layer with and without light‐absorber addition, and the normalized spectral solar irradiance density of air mass 1.5 global (AM 1.5 G) tilt solar spectrum. b) The hydrogel evaporator surface temperatures over time under one sun irradiation in the evaporated state. c) Mass change of water with hydrogel evaporators under one sun. d) The water evaporation rate and energy efficiency of hydrogel evaporators under one sun. e) Thermal conductivity of hydrogel evaporators. f) Energy distribution analysis of hydrogel evaporators.

The performance of solar vapor generation based on hydrogel evaporators is evaluated under one sun. First, light absorption capacity is an important factor affecting the rate of solar vapor generation. The bionic tree leaf layer for light absorption of the 3DP‐BHE is labeled as S200‐AC. As shown by the UV‐vis‐NIR spectra, S200‐AC with activated carbon concentration 10 mg ml^−1^ and 2 mm thick presents outstanding solar absorption characteristics (94.01%) in the 250 to 2500 nm broad wavelength range. The solar absorption of S200 without solar absorber and S200‐AC5 with 5 mg ml^−1^ solar absorber is only 35.32% and 86.03%, respectively (**Figure** **5**a and Figure S4, Supporting Information). This discrepancy should be attributed to the activated carbon particles uniformly coated on the starch skeleton improved the full‐spectrum solar absorptive capacity. Besides excellent light absorption, the photothermal conversion of the evaporator is also critical. The surface temperature of the evaporator was traced under one sun irradiation, to evaluate the photothermal conversion. When pure water evaporation is performed under one sunlight irradiation, all hydrogel evaporator surface temperatures rise in the initial 5 min and eventually stabilize at the equilibrium temperature within 20 min (Figure 5b and Figure S5, Supporting Information). The steady‐state surface temperature of 3DP‐BHE, CHE and 3D‐printed monolithic hydrogel evaporator (3DP‐MHE) are 34.2, 37.9, and 36.8°C, respectively. Freeze‐dried CHE, 3DP‐BHE and 3DP‐MHE with the same photothermal conversion layer have almost similar surface temperatures (Figure S6, Supporting Information). Thus, the slightly lower temperature of 3DP‐BHE can be attributed to the stronger cooling effect produced by evaporation.

The water evaporation rate of the 3DP‐BHE is 2.13 kg m^−2^ h^−1^, which exhibits 19% and 30% enhancement compared with the 3DP‐MHE and the CHE, respectively (Figure 5c,d). All evaporation rates were calibrated with dark‐condition evaporation data (Figure S7, Supporting Information). The energy efficiency for solar‐vapor conversion is another crucial parameter, which is calculated by Equation (1):

(1) $$\eta =\frac{\dot{m}E_{\mathrm{equ}}}{C_{\mathrm{opt}}P_{0}}$$

where $\dot{m}$ means the mass flux in steady state, C_opt_ refers to the optical concentration on the evaporation surface, and P_0_ is the solar irradiation power of one sun (1 kW m^−2^). The E_equ_ used here is the equivalent evaporation enthalpy of the water with the assistance of hydrogel evaporators (Figure S8 and Table S1, Supporting Information). Those hydrogel evaporators have the same surface composition and therefore have similar equivalent evaporation enthalpy. The solar‐to‐vapor efficiency of the 3DP‐BHE, 3DP‐MHE and CHE are 90.5%, 76.5%, and 70.1%, respectively (Figure 5d). Although the evaporation rate and solar‐to‐vapor efficiency of 3DP‐BHE are not the fastest compared with Janus ion‐selective hydrogel solar evaporator^[^ ^24^ ^]^ and hybrid hydrogel evaporator^[^ ^34^ ^]^ (Table S2, Supporting Information). However, the focus of our work is salt resistance and low cost, so we slightly sacrifice the evaporation rate to realize stable evaporation in brine, which is more important for practical application.

The main influences on evaporation rate are light absorption, water transport, and thermal isolation. All hydrogel evaporators have the same light‐absorbing layer, S200‐AC, and can achieve sufficient water transfer. Therefore, such a high evaporation rate and energy efficiency of 3DP‐BHE results from effective thermal isolation by 3D‐printed bionic structures. The thermal conductivity of 3DP‐BHE, 3DP‐MHE and CHE are 0.64, 0.83, and 0.90 Wm^−1^ K^−1^, respectively (Figure 5e). The 3DP‐MHE with the monolithic photothermal layer and the CHE without wick channels, both lead to thermal conductivity increases. This proves that the unique double‐layer bionic structure of 3DP‐BHE contributes to effective thermal isolation. The heat localization is also confirmed by equilibrium temperature infrared images of the 3DP‐BHE (Figure S9, Supporting Information). The surface temperature of the 3DP‐BHE increased rapidly in the first few minutes and gradually stabilized, while the temperature of the bulk water was maintained close to room temperature. An energy utilization analysis was performed to quantify heat losses.^[^ ^22^ ^]^ The energy distribution of the hydrogel evaporator can be expressed as:

(2) $$\dot{m}E_{equ}=\alpha P_{0}-\lambda \left(T_{1}-T_{2}\right)\frac{1}{d}-\varepsilon \sigma \left(T_{1}^{4}-T_{env}^{4}\right)-h\left(T_{1}-T_{env}\right)$$

where α represents the solar absorbance of the hydrogel evaporator, P_0_ is the solar irradiation power of one sun (1 kW m^−2^), λ is the thermal conductivity, *T*_1_ means the evaporator upper temperature, *T*_2_ is the evaporator bottom temperature, *d* is the thickness of the evaporator, ε is the emittance of the hydrogel evaporator (at thermal equilibrium, the emissivity equals the absorptivity), σ is the Stefan−Boltzmann constant (5.67×10^−8^ W m^−2^ K^−4^), *T*_env_ is the environmental temperature, *h* is the convection heat transfer coefficient (5 W m^−2^ K^−1^). The utilized of solar energy for water evaporation of the 3DP‐BHE, 3DP‐MHE and CHE are 89.1%, 76.9% and 70.9%, which is similar to solar‐to‐vapor efficiency (Figure 5f). The corresponding heat conduction losses are 2.2%, 9.7%, and 14.9%, respectively (Table S3, Supporting Information). The discrepancy in heat conduction losses is mainly due to differences in thermal conductivity induced by different structures. 3DP‐BHE with the lowest thermal conductivity can effectively block heat transfer and fully utilize solar energy for water evaporation.

### 2.5 Stable Solar Desalination Performance of the 3DP‐BHE

**Figure 6:**
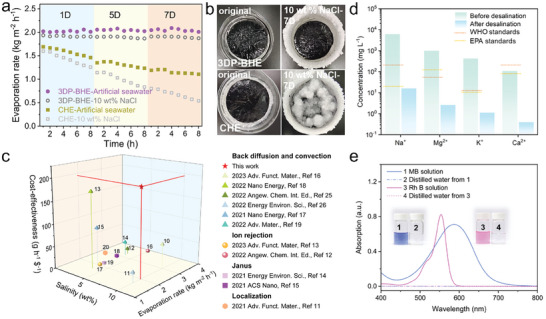
Salt rejection and desalination performance of hydrogel evaporators. a) Performance of solar desalination based on hydrogel evaporators, under one sun irradiation with seven days in brine for 8 h every day. b) Photographs of salt accumulation on the hydrogel evaporator surface after desalination in 10 wt.% NaCl for seven days. c) Cost‐effectiveness comparison with currently reported salt rejection hydrogel evaporators. d) Measured the concentrations of primary ions in artificial seawater before and after desalination using 3DP‐BHE. e) UV‐vis absorption spectra of organic dye solutions before and after purification using 3DP‐BHE.

The 3DP‐BHE maintains steady evaporation rates of 2.04 kg m^−2^ h^−1^ in artificial seawater and 1.98 kg m^−2^ h^−1^ in 10% NaCl (**Figure** **6**a). The slight reduction in evaporation rate compared with pure water is due to decreased saturated vapor pressure induced by increased salinity. The evaporation rate of the CHE decreased from 1.69 to 1.10 kg m^−2^ h^−1^ and 1.59 to 0.53 kg m^−2^ h^−1^ in artificial seawater and 10% NaCl, respectively. Similar steady evaporation rates were observed with different brine (Figure S10a, Supporting Information). The excellent salt resistance property of 3DP‐BHE can be attributed to the synergistic effect of diffusion and convection. All hydrogel evaporators enable salt ion diffusion. According to Fick's law, the salt ion diffusion rate of 3DP‐BHE is much higher than that of CHE (detailed description in Supporting Information). As for convection, the concentration of brine at the evaporation interface will continue to grow, which results in the density of brine on the evaporation surface being greater than that of bulk water. Under the effect of gravity, the high‐concentration brine on the evaporation surface will convect with the low‐concentration brine in bulk water. 3DP‐BHE with the bimodal porous structure significantly shortens the salt transportation path and reduces convective resistance. Therefore, the 3DP‐BHE assures expeditious ion convection and avoids the accumulation of salt crystals on the evaporator surface (Figure 6b and Figure S11, Supporting Information). More importantly, the cost‐effectiveness of hydrogel evaporators is an important factor that affects their practical application. Here, the cost‐effectiveness (ε) is defined as:

(3) $$\varepsilon =\frac{r}{c}$$

where r is the brine evaporation rate (kg m^−2^ h^−1^) and *c* refers to the materials cost ($ m^−2^). The interpretation of ε is how many grams of purified water can be obtained in 1 h by spending 1 USD. The raw materials cost of this 3DP‐BHE is only 10.14 $ m^−2^. With a stable evaporation rate of 1.98 kg m^−2^ h^−1^ in 10% NaCl, the 3DP‐BHE exhibits outstanding cost‐effectiveness at 195.3 g h^−1^ $^−1^ (Figure 6c and Table S4, Supporting Information), which is superior to the currently reported salt‐resistant hydrogel evaporators.

To carefully evaluate the solar desalination performance of the 3DP‐BHE, the quality of the collected water was measured using inductively coupled plasma mass spectroscopy (ICP‐MS). Figure 6d and Figure S10b (Supporting Information) show the concentration of ions before and after desalination by 3DP‐BHE. The concentrations of all four salt ions are decreased by more than three magnitudes, and the desalinated water meets the drinking water standards defined by the World Health Organization and the U.S. Environmental Protection Agency. Furthermore, we also used simulated industrial dye wastewater with methylene blue (MB) and Rhodamine B (RhB) aqueous solution to demonstrate the wastewater treatment performance of the 3DP‐BHE. As shown in Figure 6e, the dye molecule concentrations became almost zero and water became colorless after solar‐driven water evaporation, indicating potential for practical use in terms of industrial wastewater.

## 3 Conclusion

In summary, inspired by the tree transpiration and water transport porous structure, a cost‐effective 3D printed bionic hydrogel evaporator is fabricated. The bionic tree leaf for light absorption and vapor diffusion. The bionic tree trunk with the bimodal porous structure not only achieves salt ions diffusion and convection but also gives effective thermal isolation. Owing to its unique bionic structure, the 3DP‐BHE simultaneously achieves a high evaporation rate of 2.13 kg m^−2^ h^−1^ with 90.5% energy efficiency under one sun and a steady evaporation rate of 1.98 kg m^−2^ h^−1^ with impressive cost‐effectiveness of 195.3 g h^−1^ $^−1^ in 10 wt.% brine during the 7‐days desalination. Although this work provides an effective approach to low‐cost solar desalination systems, the development of a hydrogel evaporator that can withstand wave impacts and prevent microbial contamination in ocean environments remains a challenge. Future research will explore and integrate high‐strength, self‐healing and antimicrobial hydrogel evaporators for long‐term desalination without artificial intervention.

## 4 Experimental Section

### Materials

Chemicals including corn starch, activated carbon (10‐24 mesh) and nitric acid. All materials were purchased from Shanghai Aladdin Biochemical Technology Corporation and used without further purification.

### Preparation of 3D Printing Inks

For starch ink, 2 g corn starch powder was dispersed in 10 mL dilute (DI) water and stirred in a water bath at 70°C for 10 min to make the starch gelatinization, which was configured into 3D printing starch inks with starch concentrations of 200 mg ml^−1^ and named as S200 ink. For starch‐AC ink, 10 mg ml^−1^ activated carbon was added into the starch solutions before gelatinization, other steps were the same, further labeled as S200‐AC ink. Two distinct printable inks are loaded into separate syringes for use in 3D printing manufacturing.

### Preparation of 3D‐Printing Bionic Hydrogel Evaporator

3D printing was conducted using a self‐constructed 3D printer. The 3D printing requirements can be met by appropriately modifying the printing parameters (such as pressure, printed velocity, nozzle diameter, and printed trajectory). The starch inks and starch‐AC inks were contained in separate syringes fitted with an internal diameter of 300 µm stainless steel nozzles. The bottom light absorption layers were set at a height of 0.25 mm, a line width of 0.28 mm, a printing speed of 8 mm^−1^s, a cumulative stack of eight layers and 100% rectangular fill, while the water transport layers were set at a height of 0.20 mm, a line width of 0.35 mm, a printing speed of 15 mm ^−1^ s, a cumulative stack of 25 layers and 40% rectangular fill. After printing, the deposited 3D model was quickly transferred to a −20 °C freezer to fix the structure, and then five freeze‐thaw cycles were performed to obtain the final 3D printed bionic hydrogel evaporators.

### Preparation of 3D‐Printed Monolithic Hydrogel Evaporator

The 3DP‐MHE was only prepared with starch‐AC ink. The print parameters of light absorption layer are same as 3DP‐BHE. The print parameters of the water transport layer are set as a height of 0.25 mm, a line width of 0.28 mm, a printing speed of 8 mm^−1^ s, a cumulative stack of 20 layers and 40% rectangular fill. Other steps are the same as 3DP‐BHE.

### Preparation of Conventional Hydrogel Evaporator

An appropriate amount of 200 mg ml^−1^ starch and 10 mg ml^−1^ AC mix solution was introduced into the molds, then the molds were transferred to a 70°C gelatinization 10 min to form a 2 mm light absorption layer. Continue adding 200 mg ml^−1^ starch solution to the molds and 70°C gelatinization for another 10 min to form a 5 mm water transport layer. Other steps are the same as 3DP‐BHE.

### Preparation of Artificial Seawater

13.4 g NaCl, 1.13 g f MgCl_2_, 1.62 g MgSO_4_, 0.365 g KCl, 0.577 g CaCl_2_, 0.140 g NaBr, and 0.100 g NaHCO_3_ were dissolved into 500 ml DI water.

### Characterizations Methods

The rheological properties of inks were measured by rheometer (Discovery HR‐2, TA Instruments) with 25 mm diameter steel parallel‐plate geometry. The scanning electron microscope (SEM) images of freeze‐dried 3DP‐BHE were taken by JSM‐6700F, JEOL. Solar absorption (*A*) spectra was calculated by the reflectance (*R*) and transmittance (*T*) (A = 1‐R‐T), using a UV‐vis‐NIR spectrometer (Lambda 1050+) with an integrating sphere unit and automation of the reflectance measurement unit. The infrared images were implemented by a FLIR‐E5 infrared camera, with the emissivity calibrated to 0.96. Ion concentrations of artificial seawater before and after purification were all tracked by inductively coupled plasma mass spectrometry (ICP‐MS, Agilent 8900) with dilutions in 2% HNO_3_ to make the loaded ion concentration lower than 10 ppm. The dye solution and purified water absorption spectrum were conducted using UV–vis spectroscopy (Evolution 350, Thermo Fisher Scientific).

### Measurement of Solar Steam Generation

The water evaporation performance of the hydrogel evaporators was conducted in the lab with a temperature of ≈25°C and humidity ≈30% using a solar simulator (CME‐SL500, Micronergy) equipped with adjustable optical components. The solar flux was calibrated using a power meter (TES‐123) and controlled at 1000 W m^−2^ (1 sun). To reduce the influence of additional heating, the main part of the top area of the beaker was covered with a hydrogel sample of 30 mm in diameter and 7 mm in thickness, while the rest of the top area was covered with metal foil to eliminate potential errors. The evaporator samples were floated on pure water with the help of foam as the floating assistant. The mass of the water loss is measured in real‐time by a delicate lab balance (BCE224‐1CCN, Sartorius) with 0.1 mg resolution and calibrated. All evaporation rates were measured after stabilization under one sun for 60 min and calibrated with dark‐condition evaporation data.

## Conflict of Interest

The authors declare no conflict of interest.

## Supporting information

Supporting Information

## Data Availability

The data that support the findings of this study are available in the supplementary material of this article.
