# Supplementary material for: Cost‐Effective 3D‐Printed Bionic Hydrogel Evaporator for Stable Solar Desalination
Source: Adv Sci (Weinh). 2024 Feb 11;11(17):2308665. doi: 10.1002/advs.202308665 (PMC11077647; doi:10.1002/advs.202308665)
Supplement: Supplementary file 1 — Supporting Information [file ADVS-11-2308665-s001.pdf]

ADVANCED SCIENCE
Open Access

Supporting Information

for *Adv. Sci.*, DOI 10.1002/advs.202308665

Cost-Effective 3D-Printed Bionic Hydrogel Evaporator for Stable Solar Desalination

*Shuang Zhang, Meng Li, Chaorui Jiang, Dandan Zhu and Zhihui Zhang**

Supporting Information

Cost-effective 3D-printed Bionic Hydrogel Evaporator for Stable Solar Desalination

*Shuang Zhang, Meng Li, Chaorui Jiang, Dandan Zhu, Zhihui Zhang**

S. Zhang Author 1, C. Jiang Author 3, D. Zhu Author 4, Z. Zhang Author 5
Key Laboratory of Bionic Engineering, Ministry of Education, College of Biological and Agricultural Engineering, Jilin University, No. 5988 Renmin Street, Changchun 130025, People's Republic of China
E-mail: zhzh@jlu.edu.cn

M. Ling Author 2
The State Key Laboratory of Supramolecular Structure and Materials, College of Chemistry, Jilin University, No. 2699 Qianjin Street, Changchun 130023, People's Republic of China

Supporting Figures and Tables

Figure S1. The dispersion of AC particles in dilute water.
Figure S2. The mechanical strength of 3DP-BHE.
Figure S3. Water transport ability of the 3DP-BHE.
Figure S4. UV–vis–NIR spectra of the absorbent layer.
Figure S5. The infrared images of hydrogel evaporator surface temperatures in the evaporated state.

Figure S6. The infrared images of freeze-dried hydrogel evaporator surface temperatures.

Figure S7. Water evaporation rate of hydrogel evaporators under dark.

Figure S8. The evaporation enthalpy of the hydrogel evaporators.

Figure S9. The infrared images of hydrogel evaporator show the temperature distribution.

Figure S10. Salt rejection and desalination performance of 3DP-BHE.

Figure S11. Photographs of 2 g salt removal phenomenon of hydrogel evaporators.

Table S1. Comparison of evaporation enthalpy results from DSC and dark experiment.

Table S2. Comparison of evaporation rate, salt resistance and cost over currently reported hydrogel evaporators.

Table S3. Energy utilization analysis of hydrogel evaporators.

Table S4. Comparison of cost-effectiveness over currently reported salt rejection hydrogel evaporators.

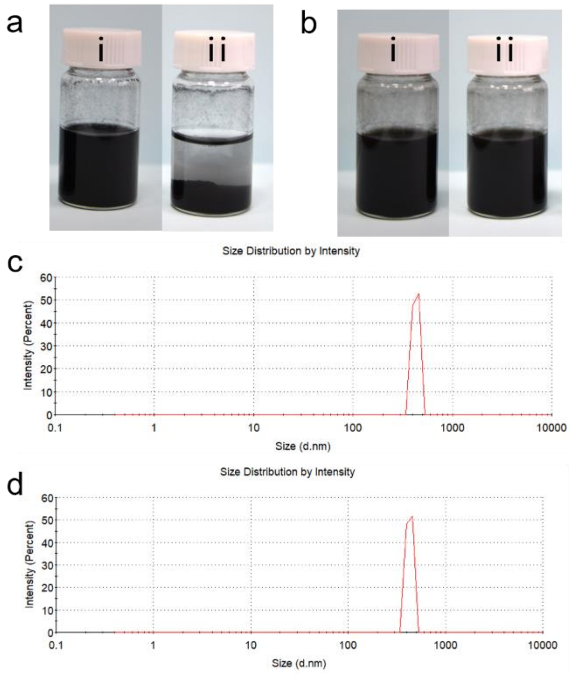

Figure S1. The dispersion of AC particles in dilute water. Photographs of different AC particles dispersed by ultrasound before and after 30 minutes of rest, a) untreated AC particles, b) hydrophilicity-treated AC particles. Dynamic light scattering measurement of different AC particle sizes, c) untreated AC particles, d) hydrophilicity treated AC particles.

Hydrophilic treatment of AC to ensure it can be uniformly dispersed in the hydrogel network. According to the method previously reported.[1] The AC power was stirred in 4% molar nitric acid for 24 hours to make their surface hydrophilic. Then the obtained AC was washed in dilute water five times and dried for about 24 hours.

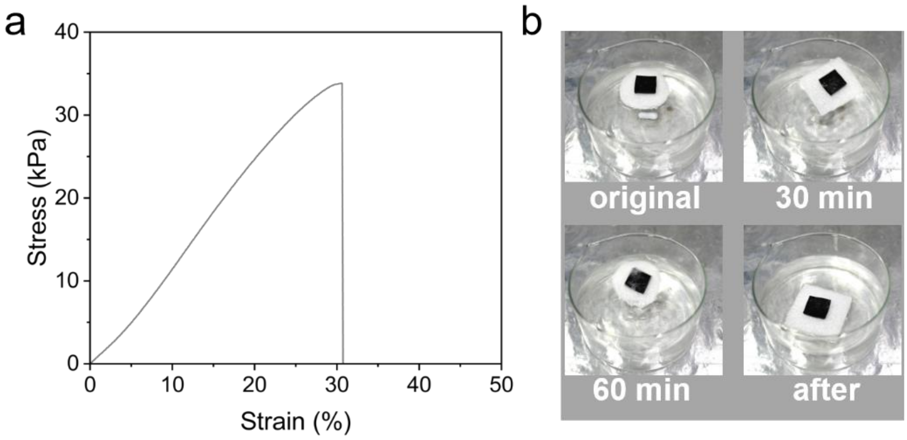

Figure S2. The mechanical strength of 3DP-BHE. a) Stress-strain curves of hydrated 3DP-BHE. b) Photographs of 3DP-BHE resisting water swirls.

As shown in **Figure S2a**, the breaking strength of hydrated 3DP-BHE reaches 32.16 ± 1.36 kPa. Using a water vortex generated by a 1000 rpm magnetic stirrer to simulate flowing seawater to test the stability of 3DP-BHE in flowing water. 3DP-BHE can maintain its bionic structure intact throughout the 60-minute test **(Figure S2b)**.

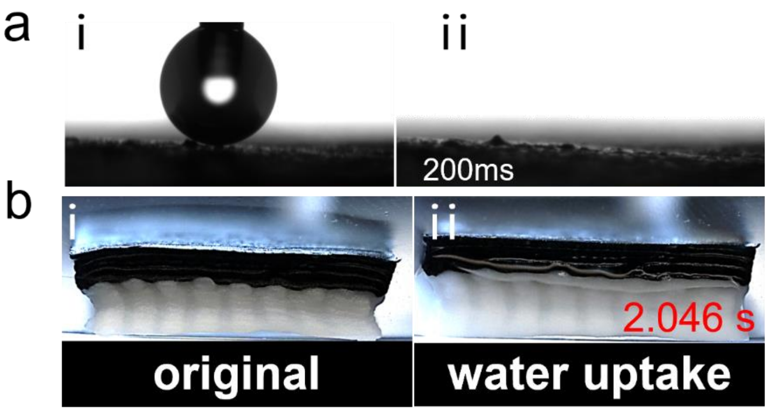

Figure S3. Water transport ability of the 3DP-BHE. a) Image of the water contact angle of 3DP-BHE. b) Screenshot of 3DP-BHE water transport speed taken by a high-speed camera

Due to the abundant hydrophilic groups of starch, 3DP-BHE displays the hydrophilic feature. Furthermore, owing to the bimodal porous structure, the 100 μl droplet penetrated the surface of 3DP-BHE in 200 ms **(Figure S3a)**. As shown in **Figure S3b**, a high-speed camera captured the transport process of 3 ml water in 3DP-BHE at a speed of 2000 frames per second. The interconnected wick channels and microchannels of 3DP-BHE continuously absorbs water and expands. Only 2.046 s, water is rapidly transported from the bottom of 3DP-BHE to the bionic leaf layer through the bionic trunk layer.

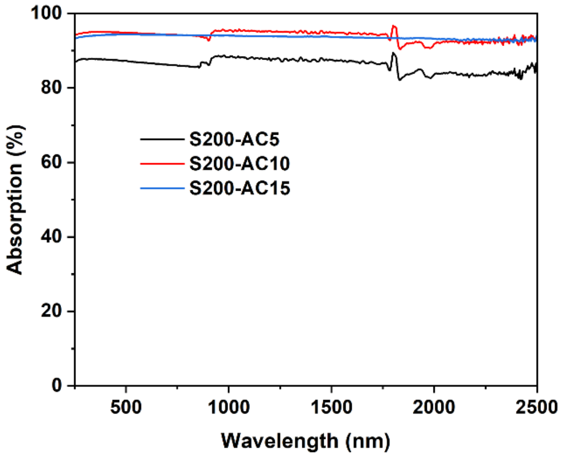

Figure S4. UV–vis–NIR spectra of the absorbent layer with different AC concentrations, fixed starch concentration 200 mg ml^{-1} and light absorbing layer thickness of 2 mm.

The bionic tree leaf layer for light absorption has an identical thickness. As shown in **Figure S4**, the light absorption of S200-AC5 (86.03%) with the activated carbon concentration of 5 mg/ml is still relatively low, while S200-AC10 (94.01%) and S200-AC15 (93.87%) exhibit efficient absorption over a broadband of the standard solar spectrum. Such results indicate that most of the solar energy can be harvested by the light-absorbing layer as long as the concentration of AC is higher than 10 mg/ml

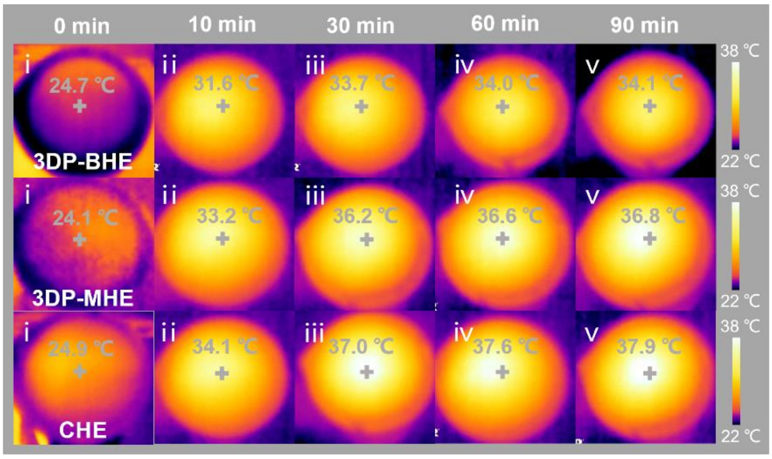

Figure S5. The infrared images of hydrogel evaporator surface temperatures over time under one sun irradiation in the evaporated state.

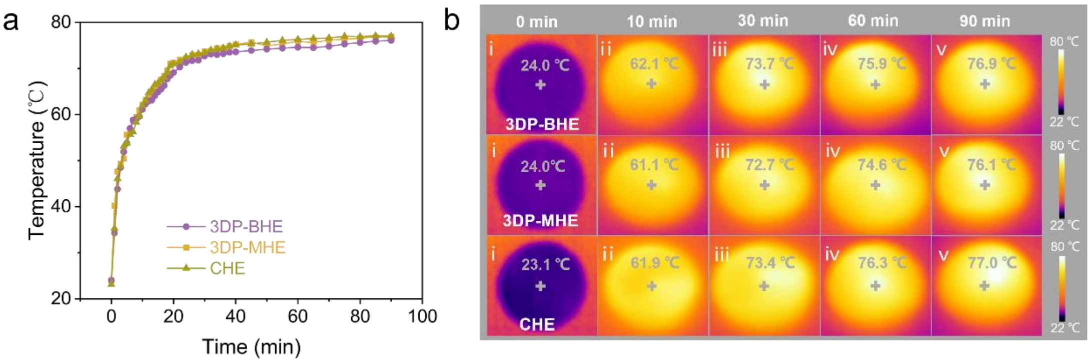

Figure S6. The infrared images of freeze-dried hydrogel evaporator surface temperatures over time under one sun irradiation. a) Surface temperatures over time, b) The corresponding infrared images.

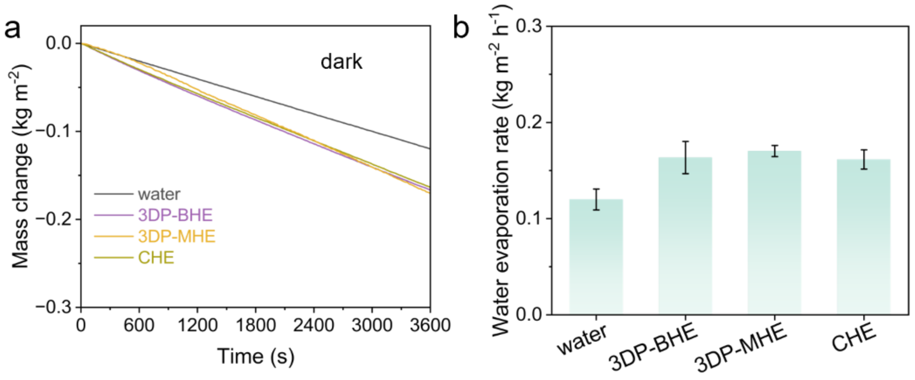

Figure S7. Water evaporation rate of hydrogel evaporators under dark. a) Mass change over time, b) water evaporation rate.

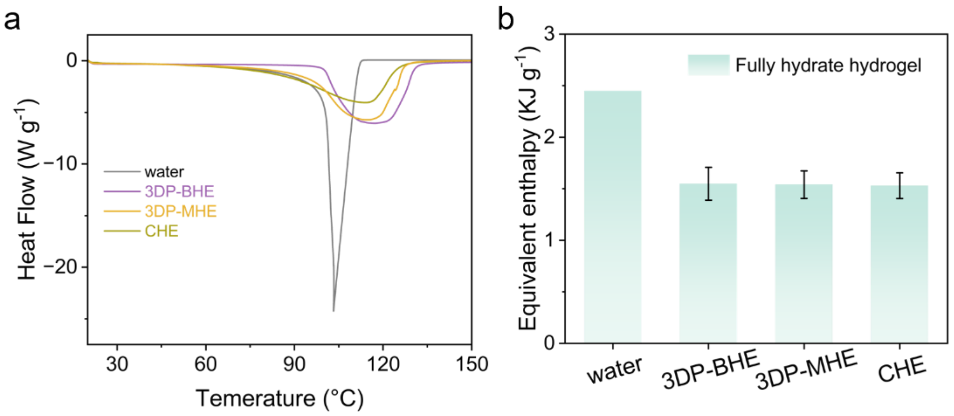

Figure S8. The evaporation enthalpy of the hydrogel evaporators. a) Differential scanning calorimetry (DSC) curves of different hydrogel evaporators. b) The calculated equivalent evaporation enthalpy of different hydrogel evaporators.

Differential scanning calorimetry (DSC) was used to confirm that the evaporation enthalpy of water in hydrogel evaporators can be reduced. The hydrogel sample was

placed in an open Al crucible and measured under nitrogen flow (50mL min^{-1}) while being heated from 20 to 160 ºC at a rate of 5 ºC min^{-1}. As shown in **Figure S3a**, the heat flow signals changed with temperature. Pure water (gray line) is a control sample, where a sharp peak of heat signal was observed, indicating the evaporation of water is completed immediately. In contrast, the water in hydrogel evaporators all had broader peaks that gradually decayed, presenting a different evaporation behavior compared with pure water. The measured vaporization enthalpy of water is 2429.3 J g^{-1} (theoretical at 2450 J g^{-1}), indicating the accuracy of the measurements. The hydrogel evaporators all showed lower vaporization enthalpy compared with pure water **(Table S1)**. In this case, the DSC measurements can only qualitatively evaluate the reduction of vaporization enthalpy by hydrogel evaporators. This is because DSC presents a full dehydration process of hydrogels, which involves the energy requirement of vaporizing all three types of water (bound, intermediate, and free).

Since hydrogel evaporators are at their fully or nearly fully hydrated state during the solar vapor generation process, the more accurate evaporation enthalpy should only involve free water and intermediate water. As such, a designed experiment was conducted to observe and estimate this evaporation enthalpy of hydrogel evaporators, which presents a slight dehydration process of hydrogel evaporators. Pure liquid water and different hydrogel evaporators with the same surface area were synchronously located in a closed desiccator with supersaturated potassium carbonate solution under room temperature with ambient air pressure. The mass changes were recorded to estimate the evaporation enthalpy of different hydrogel evaporators by making a comparison with the known theoretical value of liquid water, 2450 J g^{-1}, using identical power input (U_{in}).

$$U_{in} = E_{equ} m_g = E_0 m_0 \quad (1)$$

where m_g is the mass change of different hydrogel evaporators under dark condition, E_0 and m_0 refers to the evaporation enthalpy and mass change of bulk water within the same environmental condition, respectively. At least five measurements were

performed using samples from different synthesized batches to obtain the average value. The obtained equivalent evaporation enthalpy of water in hydrogel evaporators was reduced a lot compared to that of the pure water as well as in the same trend with DSC results **(Table S1)**. The equivalent evaporation enthalpy estimated here was used to calculate energy efficiency.

Table S1. Comparison of evaporation enthalpy results from DSC and dark experiment.

Enthalpy (J g^{-1})	Pure water	BHE	3DP-BHE	3DP-BHE-M
DSC	2429.3	1824.5	1804.9	1864.3
Dark experiment	2450	1549.9659±88.6954	1530.3910±98.0123	1540.3982±89.2123

Table S2. Comparison of evaporation rate, salt resistance and cost over currently reported hydrogel evaporators.

Ref	Solar evaporators	Total cost ($ m^{-2})	Evaporation rate (kg m^{-2} h^{-1}. under one sun)	salt resistance continuous evaporation time
This work	3D-printed bionic hydrogel evaporator	10.1379	1.98 (10 wt% NaCl)	7 days with 8 h per day for 10 wt% NaCl
[2]	Janus ion-selective hydrogel solar evaporator	928.15	6.86 (15 wt% NaCl)	7 days with 6h per day for 15 wt% NaCl
[3]	Pvbips/PPy high-efficient brine evaporator	453.8	3.17 (3.5 wt% NaCl)	8h for 3.5 wt% NaCl
[4]	3D hydrogel evaporator with an envisaged vertical radiant structure	236.43	3.52 (20 wt% NaCl)	7 days with 8 h per day for 20 wt% NaCl
[5]	hierarchically porous radiation-absorbing hydrogel film	173.3245	1.983 (4 wt% NaCl)	6 h
[6]	radiation-absorbing hydrogel film	99.93	2.11 (25 wt% NaCl)	140 h for 25 wt% NaCl
[7]	light-absorbing sponge-like hydrogel	293.21	3.6 (pure water)	48 h for seawater
[8]	hierarchically nanostructured gel	98.83	3.2 (pure water)	28 days for seawater

[9]	hybrid hydrogel evaporator	14.9	3.2 (pure water)	NA
[10]	$Ti_3C_2T_x$ MXene/rGO hybrid hydrogel evaporator	556.13	3.62 (pure water)	NA
[11]	biomimetically assembled 3D sponge-like hydrogel	14497.56	3.45 (pure water)	NA

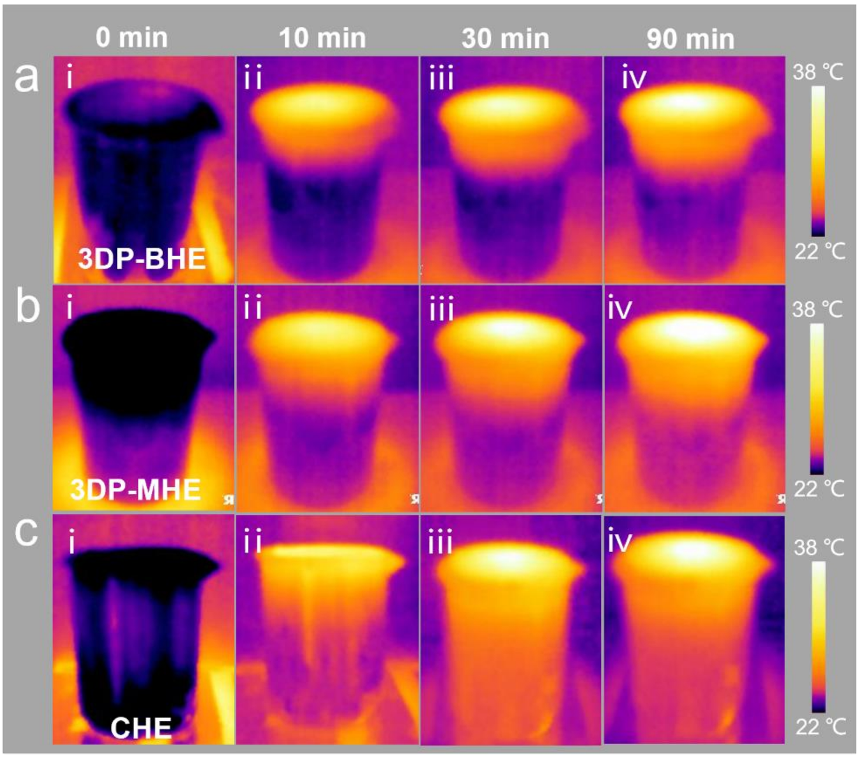

Figure S9. The infrared images of hydrogel evaporator show the temperature distribution after exposure to one sun irradiation of 0 min, 10 min, 30 min, and 90 min. a) 3DP-BHE, b) 3DP-MHE, c) CHE.

Table S3. Energy utilization analysis of hydrogel evaporators.

	Reflection (%)	water evaporation (%)	Conduction (%)	Convection (%)	Radiation (%)
3DP-BHE	6.0	89.1	2.2	1.2	1.5
3DP-MHE	6.0	76.9	9.7	3.3	4.1
CHE	6.0	70.9	14.9	3.6	4.5

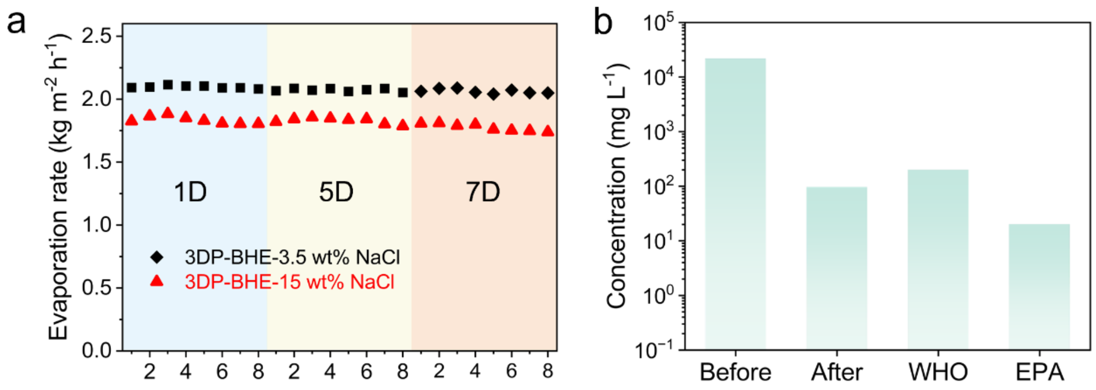

Figure S10. Salt rejection and desalination performance of 3DP-BHE. a) 3DP-BHE desalinate different brines. b) The Na^+ ion concentration before and after 10 wt% NaCl desalination with 3DP-BHE.

As shown in **Figure S10a**, the evaporation rates of 3DP-BHE remained at 2.06 kg m^{-2} h^{-1} and 1.77 kg m^{-2} h^{-1} after 7 days of continuous desalination of 3.5 wt% and 10 wt% NaCl, respectively. This proves that the 3DP-BHE has outstanding salt resistance and stability, and therefore holds great promise for practical solar desalination of hypersaline waters. After 10 wt% NaCl desalination, the concentration of Na^+ decreased from 3.6×10^4 mg L^{-1} to 9.6×10^1 mg L^{-1}, which was still lower than the 2.0×10^2 mg L^{-1} specified by WHO **(Figure S10b)**.

Salt resistance mechanism

Diffusion flux can be calculated by Fick's law:

$$J = -D\frac{dc}{dx} = -D\frac{c_{bulk\,water} - c_{surrface}}{\tau L} \quad (2)$$

where J is the diffusion flux, the negative sign indicates that the diffusion direction is opposite to the concentration gradient direction, and D means the diffusion coefficient (1.60×10^{-9} m^2 s^{-1}), $c_{bulk\ water}$ and $c_{surface}$ respectively represent the salt concentration of the bottom water and the evaporation interface, τ is the tortuosity of the salt transport channel. For the 3DP-BHE with the wick channels, τ is approximately equal to 1. For

the CHE, high tortuosity makes τ far greater than 1. At the same thickness, the salt ion diffusion rate of 3DP-BHE is much larger than that of CHE.

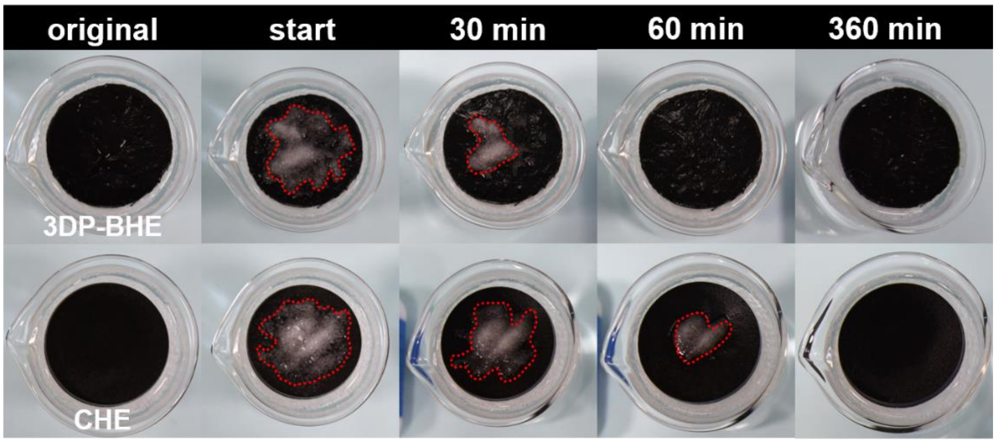

Figure S11. Photographs of 2 g salt removal phenomenon of hydrogel evaporators.

The bionic bimodal porous structure of 3DP-BHE accelerates the convection and diffusion of salt ions. As shown in **Figure S11**, the NaCl crystals were eliminated in 60 min after loading on the surface of 3DP-BHE, property much faster than CHE.

Table S4. Comparison of cost-effectiveness over currently reported salt rejection hydrogel evaporators.

Ref	Raw Materials	Estimated single item cost ($ m^{-2})	Total cost ($ m^{-2})	Salinity (wt%)	Evaporation rate (kg m^{-2} h^{-1})	Cost-effectiveness (g h^{-1} $\$^{-1}$)
**Back diffusion and convection**						
This work	-Starch<br>-Activated carbon<br>-Nitric acid	-9.54<br>-0.29<br>-0.01	10.14	10	1.98	195.3
[12]	-Kevlar para-aramid pulp<br>-polyvinyl alcohol<br>-DMSO<br>-pyrrole<br>-FeCl3	-5.06<br>-44.9<br>-95.2<br>-57.3<br>-65.6	53.61	10	2.99	55.8
[13]	-Starch<br>-acrylamide<br>-ammonium persulfate<br>-BIS	-8.41<br>-43.60<br>-0.1747<br>-21.86	74.05	10	1.65	22.28

[14]	-MoS_2<br>-acrylamide<br>-BIS<br>-TMEDA<br>-sodium dodecyl sulfate<br>-ammonium persulfate	-128.78<br>-47.68<br>-35.41<br>-52.95<br>-0.38<br>-4.86	270.06	3.5	3.297	12.21
[15]	-APS<br>-Pyrrole<br>-Water<br>-NaOH<br>-BCH<br>-Electricity	-1.38<br>-6.77<br>-0.043<br>-1.06<br>-0.6<br>-0.06	9.98	3.5	1.7	171.34
[16]	-PVA<br>-Petroleum coke<br>-$NaNO_3$<br>-H_2SO_4<br>-$KMnO_4$<br>-H_2O_2	-26.14<br>-0.68<br>-2.71<br>-44.61<br>-14.17<br>-14.60	102.91	3.5	3.18	30.80
[17]	-Sodium Molybdate Dihydrate<br>-Thiourea<br>-Sodium Alginate<br>-Melamine foam	-10.76<br>-7.18<br>-1.28<br>-2.65	21.87	3.5	1.92	87.79
**Ion rejection**						
[1]	-[2-(methacryloyloxy)ethyl]dimethyl-(3-sulfopropyl)ammonium hydroxide<br>-ammonium persulfate<br>-PEGDA<br>-TMEDA<br>-activated carbon pellets	-2617.375<br>-8.9375<br>-13.5625<br>-1.625<br>-0.875	2642.375	5	3.88	1.46
[18]	-sulfobetaine methacrylate<br>-BIS<br>2-hydroxy-2-methylpropiophenone<br>-DMSO<br>-APS<br>-Pyrrole	-1126.95<br>-2.765<br>-0.284<br>-95.15<br>-45.29<br>-424.56	1695	3.5	2.024	1.19
**Janus**						
[19]	-Flake graphite<br>-H_2SO_4<br>-H_2O_2<br>-$KMnO_4$	-0.0547<br>-301.78<br>-73.81<br>-1.05	492.82	3.5	2.78	5.64

	-ammonia	-1.64				
	-PVA	-109.84				
	-SDS	-4.65				
	-chitosan	-2.50				
	-glacial acetic acid	-0.168				
	-PVA	-0.0919				
[20]	-ethanol	-45.78	2949.5	3.5	2.21	0.749
	-acetic anhydride	-44.42				
	-copper foam	-1107				
	-copper foil	-1749.5				
**Localization**						
	-lithium fluoride	-0.13				
	-HCl	-0.24				
	-MAX (Ti_3AlC_2)	-5.84				
[21]	-fiber cloth	-0.1367	95.07	3.5	2.26	23.77
	-PVA	-88.27				
	-glutaraldehyde	-0.45				

The bionic hydrogel evaporator, 3DP-BHE, was made with low cost in mind. For corn starch powder, the price is \$ 11.9 kg^{-1} (Shanghai Aladdin Biochemical Technology Corporation). Here, ~800 g starch was needed for 1 m^2 of the 3DP-BHE. Therefore, the cost of the starch part is \$ 9.84 m^{-2}. Activated carbon powder stands out with the lowest market price, \$ 14.5 kg^{-1} (Shanghai Aladdin Biochemical Technology Corporation). The total cost of AC is 0.29 \$ m^{-2}. The cost of hydrophilic treatment of AC with nitric acid is 0.01 \$ m^{-2}. The overall cost of 3DP-BHE in 1 m^2 is estimated to be \$ 10.14 m^{-2}, which favors its large-scale applications and potential for commercialization. The cost-effectiveness (ε) was defined to be the different concentration brine evaporation rate (kg m^{-2} h^{-1}) per cost (\$ m^{-2}), which can be understood as how many grams of purified water can be received in one hour if spend one U.S. dollar. Compared to previously reported evaporators, this work (195.3 g h^{-1} $\$^{-1}$) outperformed most of the currently reported solar evaporators in terms of cost-effectiveness **(Table S4)**.

References

[1] C. X. Lei, W. X. Guan, Y. H. Guo, W. Shi, Y. Y. Wang, K. P. Johnston, G. H. Yu, *Angew. Chem. Int. Ed.* **2022,** *61*, e202208487.

[2] N. He, Y. Yang, H. Wang, F. Li, B. Jiang, D. Tang, L. Li, Adv. Mater. **2023,** *35*, 2300189.

[3] S. Y. Zheng, J. Zhou, M. Si, S. Wang, F. Zhu, J. Lin, J. Fu, D. Zhang, J. Yang, *Adv. Funct. Mater.* **2023,** 2303272.

[4] X. Liu, F. Chen, Y. Li, H. Jiang, D. D. Mishra, F. Yu, Z. Chen, C. Hu, Y. Chen, L. Qu and W. Zheng, *Adv. Mater.* **2022,** *34*, 2203137.

[5] S. Meng, X.-J. Zha, C. Wu, X. Zhao, M.-B. Yang, W. Yang, Nano Lett. **2021,** *21*, 10516.

[6] L. Zhang, Y. Zhang, M. Zou, C. Yu, C. Li, C. Gao, Z. Dong, L. Wu, Y. Song, *Adv. Funct. Mater.***2023,** *33*, 2300318.

[7] Y. Guo, X. Zhou, F. Zhao, J. Bae, B. Rosenberger and G. Yu, ACS Nano **2019,** *13*, 7913.

[8] F. Zhao, X. Zhou, Y. Shi, X. Qian, M. Alexander, X. Zhao, S. Mendez, R. Yang, L. Qu, G. Yu, Nat. Nanotechnol. **2018,** *13*, 489.

[9] Y. Guo, H. Lu, F. Zhao, X. Zhou, W. Shi, G. Yu, *Adv. Mater.* **2020,** *32*, 1907061.

[10] Y. Lu, D. Fan, Y. Wang, H. Xu, C. Lu, X. Yang, ACS Nano **2021,** *15*, 10366.

[11] C. Lei, J. Park, W. Guan, Y. Zhao, K. P. Johnston, G. Yu, *Adv. Funct. Mater.* **2023,** *33*, 2303883.

[12] H. Li, W. X. Zhang, J. W. Liu, M. Z. Sun, L. Wang, L. Z. Xu, *Adv. Funct. Mater.* **2023**, 2308492.

[13] X. J. Mu, J. H. Zhou, P. F. Wang, H. Chen, T. T. Yang, S. Y. Chen, L. Miao, T. Mori, *Energy Environ. Sci.* **2022**, *15*, 3388.

[14] P. Liu, Y. B. Hu, X. Y. Li, L. Xu, C. Chen, B. L. Yuan, M. L. Fu, *Angew. Chem. Int. Ed.* **2022,** *61*, e202208587.

[15] Z. Yu, R. N. Gu, Y. X. Zhang, S. Guo, S. A. Cheng, S. C. Tan, *Nano Energy* **2022,** *98*, 107287.

[16] H. Q. Zou, X. T. Meng, X. Zhao, J. S. Qiu, *Adv. Mater.* **2023,** *35*, 2207262.

[17] J. X. Xiao, Y. Guo, W. Q. Luo, D. Wang, S. K. Zhong, Y. R. Yue, C. N. Han, R. X. Lv, J. B. Feng, J. Q. Wang, W. Huang, X. L. Tian, W. Xiao, Y. J. Shen, *Nano Energy* **2021,** *87*, 106213.

[18] B. L. Peng, Q. Lyu, M. M. Li, S. Du, J. T. Zhu, L. B. Zhang, *Adv. Funct. Mater.* **2023,** *33*, 2214045.

[19] C. Ma, Q. L. Liu, Q. Q. Peng, G. H. Yang, M. Jiang, L. Zong, J. M. Zhang, *Acs Nano* **2021,** *15*, 19877.

[20] H. Z. Yao, P. P. Zhang, C. Yang, Q. H. Liao, X. Z. Hao, Y. X. Huang, M. Zhang, X. B. Wang, T. Y. Lin, H. H. Cheng, J. Y. Yuan, L. T. Qu, *Energy Environ. Sci.* **2021**, *14*, 5330.

[21] L. Li, N. He, B. Jiang, K. W. Yu, Q. Zhang, H. T. Zhang, D. W. Tang, Y. C. Song, *Adv. Funct. Mater.* **2021,** *31*, 2104380.
